# Aflatoxin B1 Toxicity in Zebrafish Larva (*Danio rerio*): Protective Role of *Hericium erinaceus*

**DOI:** 10.3390/toxins13100710

**Published:** 2021-10-08

**Authors:** Davide Di Paola, Carmelo Iaria, Fabiano Capparucci, Marika Cordaro, Rosalia Crupi, Rosalba Siracusa, Ramona D’Amico, Roberta Fusco, Daniela Impellizzeri, Salvatore Cuzzocrea, Nunziacarla Spanò, Enrico Gugliandolo, Alessio Filippo Peritore

**Affiliations:** 1 Department of Chemical, Biological, Pharmaceutical, and Environmental Science, University of Messina, 98166 Messina, Italy; davide.dipaola@unime.it (D.D.P.); carmelo.iaria@unime.it (C.I.); fabiano.capparucci@unime.it (F.C.); rsiracusa@unime.it (R.S.); rdamico@unime.it (R.D.); rfusco@unime.it (R.F.); dimpellizzeri@unime.it (D.I.); aperitore@unime.it (A.F.P.); 2 Department of Biomedical and Dental Sciences and Morphofunctional Imaging, University of Messina, 98166 Messina, Italy; cordarom@unime.it; 3 Department of Veterinary Science, University of Messina, 98166 Messina, Italy; rcrupi@unime.it (R.C.); egugliandolo@unime.it (E.G.); 4 Department of Pharmacological and Physiological Science, Saint Louis University School of Medicine, Saint Louis, MO 63130, USA

**Keywords:** aflatoxin B1, oxidative stress, *Hericium* *erinaceus*

## Abstract

Aflatoxin B1 (AFB1), a secondary metabolite produced by fungi of the genus Aspergillus, has been found among various foods as well as in fish feed. However, the effects of AFB1 on fish development and its associated toxic mechanism are still unclear. In the present study, we confirmed the morphological alterations in zebrafish embryos and larvae after exposure to different AFB1 doses as well as the oxidative stress pathway that is involved. Furthermore, we evaluated the potentially protective effect of *Hericium* erinaceus extract, one of the most characterized fungal extracts, with a focus on the nervous system. Treating the embryos 6 h post fertilization (hpf) with AFB1 at 50 and 100 ng/mL significantly increased oxidative stress and induced malformations in six-day post-fertilization (dpf) zebrafish larvae. The evaluation of lethal and developmental endpoints such as hatching, edema, malformations, abnormal heart rate, and survival rate were evaluated after 96 h of exposure. *Hericium* inhibited the morphological alterations of the larvae as well as the increase in oxidative stress and lipid peroxidation. In conclusion: our study suggests that a natural extract such as *Hericium* may play a partial role in promoting antioxidant defense systems and may contrast lipid peroxidation in fish development by counteracting the AFB1 toxicity mechanism.

## 1. Introduction

Aquaculture is a key aspect of the world food business, supplying about half of all seafood intended for human consumption. The use of plant-based proteins instead of fishmeal in commercial feed formulations has acquired general favor in recent years [1]. However, a growing reliance on plant-based ingredients has raised the potential of mycotoxins being introduced into feed during manufacturing and storage [2]. Mycotoxins are posing a growing hazard to aquaculture, with aflatoxin, one of these mycotoxins, posing the greatest risk to farm-raised fish. Aflatoxin B1- (AFB1) contaminated feed has immediate detrimental effects on the health of farmed fish, but it also raises the possibility of pollutants being passed down the food chain to consumers [3]. The aflatoxins, secondary metabolites of the molds *Aspergillus flavus*, *Aspergillus parasiticus*, and, to a lesser degree, *Aspergillus nomius*, are a class of compounds linked to pyranocoumarin. [4]. Several investigations have revealed that Aspergillus and AFB1 contamination in fish feed is widespread across the world. Aspergillus is the most common species that can be isolated from fish feeds in South America, from about 60 to 70%, while AFB1 is detected in 55% of complete fish feeds [2]. In the United States and Canada, losses due to the ingestion of mycotoxin-contaminated feeds are high, about USD 5 billion worth of animals. In Asia and Africa, the mean levels of AFB1 that were found were high compared, for example, to Europe, where these levels were negligible (0.43 μg/kg). In fact, in Asia, average levels were found to range from = 51.83 μg/kg, with a maximum of 220.61 μg/kg, while in Iran, higher AFB1 levels were found in fishmeal (average = 67.4 µg/kg) compared to those found in the wheat (average = 12.4 µg/kg), wheat flour (average = 2.3 µg/kg), and starch (average = 1.8 µg/kg) used to feed fish [3,5,6,7,8]. AFB1, which is considered to be a toxic xenobiotic, has shown several adverse effects on human and animal health, including hepatocarcinogenic, mutagenic, and genotoxic effects [9].

Several studies have shown how AFB1 can cause increased oxidative stress when ingested in excessive amounts and repeatedly in different mouse animal models [10]. In recent years, the mechanisms of AFB1 immunotoxicity have been extensively described, and several reports have suggested that its pro-oxidation ability is the principal pathological basis for AFB1 toxicity. In the metabolic processing of AFB1, the alteration of glutathione (GSH) and glutathione peroxidase (GPx), superoxide dismutase (SOD), catalases (CAT), and glutathione S-transferase (GST) could disturb the cellular redox balance and thus result in oxidative stress-induced multiple tissue injury [11,12]. Reactive intermediates produced by oxidative stress can cause oxidative damage that affects cell membranes, lipoproteins, and other molecules, leading to lipid peroxidation [13]. The degradation products of lipid peroxides, mostly aldehydes such as malonaldehyde (MDA), have received a lot of attention because they are the most reactive compounds [13,14,15]. Thus, the neutralization of stress oxidative stress and the inhibition of apoptosis are believed to be potent targets for protection against AFB1-induced immunotoxicity [16,17].

In recent years, numerous studies have been conducted on the potential antioxidant actions of bioactive compounds, encouraging their use in both the maintenance of human health and for the prevention of disease by supplementation in aquafeed. In particular, *Hericium erinaceus* is among the most characterized medicinal mushrooms and focuses on the nervous system [18]. The antioxidative activity of *Hericium erinaceus* has been demonstrated in several pre-clinical experimental models of neurodegenerative disorders such as epilepsy, Alzheimer’s disease (AD), traumatic brain injury (TBI), and Parkinson’s disease [18,19,20]. Moreover, previous studies on zebrafish embryos have shown the protective and repairing effects of *Hericium erinaceus* in ethanol-induced neuronal loss [21]. Zebrafish (*Danio rerio*) has been well accepted as a general fish and vertebrate model to investigate chemical toxicity and the associated mechanisms because of its easy ability to be bread in the laboratory, great fecundity, fast development, short life cycle, and eggs and embryo transparency [22]. Therefore, it is an outstanding vertebrate model due to its high developmental similarity to mammals [10,23]. Because of these reasons, toxicity evaluation on zebrafish can serve as a meaningful reference not only for the humans but also for fish safety [24].

The objective of this study was to evaluate the toxicity of AFB1 on a zebrafish model, addressing lethal effects and sub-lethal endpoints related to embryonic and larval development, and also assessed the molecular mechanisms of AFB1 to induce oxidative stress and apoptosis. Furthermore, it was investigated the potentially protective effect of natural compound *Hericium* to contrast oxidative damage AFB1 exposure induced.

## 2. Results

### 2.1. Viability and Morphology of Zebrafish Embryos after AFB1 Treatment

**Figure 1:**
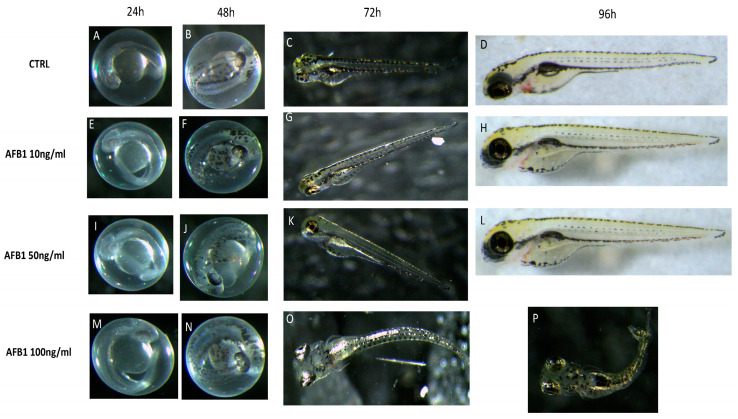
The morphological abnormalities in zebrafish caused by AFB1 exposure. Optical micrographs (**A**–**P**) showing physical malformation in comparison with controls after incubation with AFB1 for 96 hpf. No malformations were observed in low doses of AFB1 after 24 hpf (**E**,**I**), 48 hpf (**F**,**J**), 72 hpf (**G**,**K**), and 96 hpf (**H**,**L**) and in high dose of AFB1 after 24 hpf (**M**) and 48 hpf (**N**). The malformations were observed for high doses of AFB1 after 72 hpf (**O**) and 96 hpf (**P**). (**A**–**D**) represent the normal development of Zebrafish embryos. Images were taken from lateral view under a dissecting microscope (magnification 25). Scale bar, 500 mm.

In order to identify the suitable concentrations and time points for the following experiments, AFB1 concentrations of 10, 50, and 100 ng/mL were added into embryo water and were applied to observe the larvae morphology until 96 hpf. As presented in Figure 1, the AFB1 concentrations of 10 and 50 ng/mL did not alter the zebrafish morphology after 96 hpf compared to the control group (Figure 1).

The AFB1 100 ng/mL group induced a suite of abnormalities, including body axis curvature, pericardial edema, and large yolk sac, in the zebrafish embryos (Figure 1). The curvature was observed in the zebrafish exposed to AFB1 at concentrations as low as 100 ng/mL, and this peculiar phenotype was observable in 80% of larvae at the AFB1 concentration of 100 ng/mL.

### 2.2. Survival, Heart and Hatching Rate of Zebrafish Embryos after AFB1 Treatment

**Figure 2:**
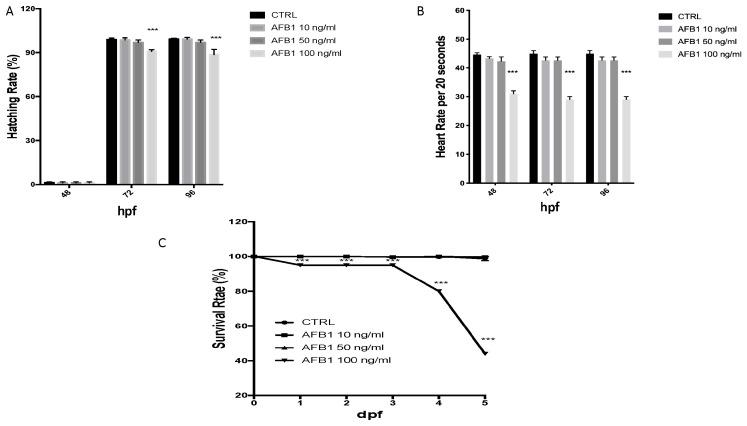
The hatching (**A**), heart (**B**) and survival (**C**) rate of embryos was determined at the designated time. The asterisk represents a statistically significant difference when compared to the controls: *** at *p* < 0.001 level.

The effect of AFB1 on embryo development was observed for up to 96 hpf. Embryo development in the control was normal: hatching began at 72 hpf, and mortality was low (Figure 2 A–C). When the embryos were treated with various concentrations of AFB1 (10, 50, and 100 ng/mL), the hatching rate decreased significantly in the high concentration group. Compared to the control, for the embryos exposed to low concentrations (10 and 50 ng/mL), no hatching influence was found. The hatching delay occurred after 72 hours with the AFB1 treatment with a concentration of 100 ng/mL. By 96 hpf, around 10% of the the remaining embryos from the AFB1 groups at with the 100 ng/mL concentration treatment that had not hatched.

Heart rates were recorded to determine the effect of AFB1 on cardiac function. In both the control and AFB1 10 ng/mL-treated embryos as well as in the 50 ng/mL group, there were not differences in the heart rate from 48 to 96 hpf. However, from 48 to 96 hpf, significant bradycardia was observed in the embryos treated with 100 ng/mL AFB1 compared to the controls (Figure 2B).

Lethality was caused right from 1 to 3 dpf at 100 ng/mL and in the following days. Neverthless, no mortality had occurred in the embryos who were treated with concentrations lower than 10 and 50 ng/mL by 6dpf. The AFB1-induced zebrafish mortality curve is presented in Figure 2C.

### 2.3. Effect of AFB1 on Lipid Peroxidation and Stress Oxidative Pathway and Hericium Protective Action

**Figure 3:**
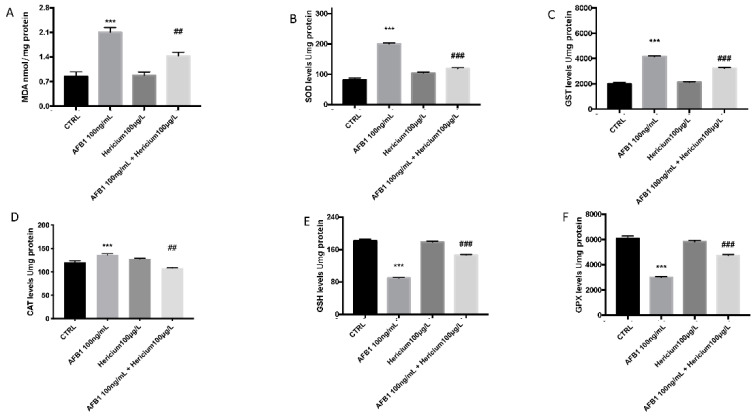
Effects of AFB1 exposure on the MDA (**A**); activity of SOD (**B**), GST (**C**) CAT (**D**), GSH (**E**), and GPx (**F**) in the larval zebrafish. Embryonic zebrafish were exposed to AFB1, *Hericium,* and both together for 96 hpf. Data are expressed as the mean ± SEM of four replicates (about 20 larvae per replicate). The asterisk represents a statistically significant difference when compared to the controls: *** at *p* < 0.001 level; ^##^ at *p* < 0.01 level; ^###^ at *p* < 0.001 level.

The results in Figure 3 illustrate that the MDA levels in the larval zebrafish increased significantly after exposure to AFB1 100 ng/mL at 96 h compared to the control group. However, there was an inhibition of the MDA levels increased after AFB1 exposure following the treatment of *Hericium* compared to the AFB1 group (Figure 3A). Correspondingly, the GSH content as well as the GPx levels decreased significantly in the larvae after exposure to AFB1, while SOD, CAT, and GST increased in presence of AFB1 (Figure 3B–D) compared to the control. *Hericium* treatment in the larvae after AFB1 exposure decreased the levels of MDA, SOD, CAT, and GST and at the same time, was able to increase the level of GSH and GPx compared to the AFB1 100 ng/L group (Figure 3E,F).

### 2.4. Hericium Preventive Effect on AFB1 Induced Apoptotic Process in Zebrafish Larvae

**Figure 4:**
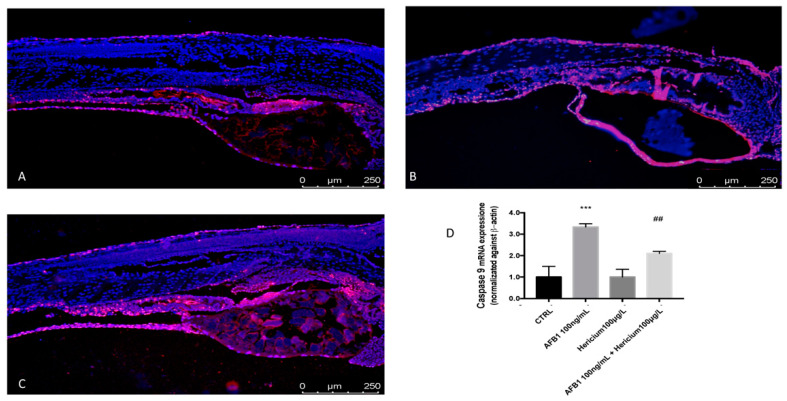
TUNEL assays indicated an abnormal apoptotic pattern (red fluorescence) in zebrafish embryos treated with AFB1 100 ng/mL at 96 hpf. CTRL(**A**) AFB1 100 ng/mL (**B**) and AFB1 100 ng/mL + *Hericium* 100 μg/L (**C**). mRNA expression of apoptosis-related genes caspase-9 (**D**). The asterisk represents a statistically significant difference when compared to the controls: *** at *p* < 0.001 level; ## at *p* < 0.01 level.

To investigate if apoptosis was induced in zebrafish embryos upon exposure to AFB1, TUNEL assays were performed to detect apoptotic cells at 96 hpf. In embryos treated with AFB1 100 ng/mL, a high level of apoptosis was detected in the yolk, trunk, and tail, whereas few labeled cells were observed in the control embryos (Figure 4A,B). Treatment with *Hericium* was able to reduce apoptosis in terms of labeled cells after AFB1 exposure (Figure 4C). To assess whether AFB1 induced apoptosis, the expression levels of caspase-9 expression was also examined. The caspase-9 levels were significantly upregulated at 96 hpf after AFB1 100 ng/mL treatment, and *Hericium* was able to reduce apoptotic levels of caspase-9 at 96 hpf after AFB1 intoxication (Figure 4D).

## 3. Discussion

One of the major issues for aquaculture productivity is the presence of mycotoxins in feed [25]. However, there is no paper discussing the level of aflatoxins in surface water to date due to their hydrophobic character. In this study, we used aflatoxin concentrations close to those found in feed and that are known to be toxic based on studies that were previously performed during the early stages of embryonic development in zebrafish larvae [10,26]. Several studies have shown how aflatoxin can cause increased oxidative stress when ingested in excessive amounts and repeatedly in fish models [27,28,29].

Early developmental exposure of zebrafish larvae to AFB1 may result in developmental alterations such as curvature of the body axis or edema formation in the yolk sac or pericardium. Previous studies have shown that exposure to AFB1 alone or in combination with other mycotoxins in zebrafish embryos can cause toxicity in zebrafish embryos [30,31] as well as behavioral alterations in terms of average velocity and the movement ratio, and can also create neurotoxicity and increase liver size; however, no significant malformations have been shown [29,32].

In accordance with previously conducted studies [29,31] where AFB1 was shown to be teratogenic, increasing the incidence of malformations such as spinal lordosis, pericardial edemas, narrowed head, elongated heart among others, in the present study, we observed that AFB1 exposure strongly affected the development of the embryos. The toxic action of AFB1 is often associated with increased oxidative stress [10,33,34,35,36]. The mechanism of oxidative stress is regulated by the balance of the oxidant–antioxidant ratio. In fact, the activation of the antioxidant mechanism of the organism serves to compensate for the excessive production of ROS, which attacks proteins, membrane phospholipids, and nucleic acids [37]. Fish have specific systems to protect themselves from the damage of oxidative stress Fish have unique systems for protecting themselves against damaging oxidative effects, which work by evaluating the antioxidant enzymes [38,39]. Our results showed a decrease in the GSH and GPx levels after AFB1 exposure. GPx mainly catalyzes the reduction of H2O2 but at the expense of GSH [40]. We thought that the decrease in the GPx levels may be due to GSH depletion induced by AFB1 [41]. The decreased levels of GSH after AFB1 exposure are in accordance with previous studies that reported a depletion of GSH levels in an early life stage zebrafish model. In a previous study, *Hericium* was able to increase levels of both GPx and GSH, which were decreased following the damage induced by scopolamine in zebrafish, as was the case in our study.

However, in the early stages of diseases, as a compensatory action to manage a harmful invader, antioxidant enzymes can be over-expressed [28]. In fact, the expression of enzymes and molecules that are normally involved in the antioxidant mechanism does not always respond in the same way after damage. Thus, the increases of SOD and CAT as well as GST could be explained by the self-protection mechanism for antioxidative stress in fish [38]. In the present study, CAT, SOD, and GST levels were significantly higher than in the control group after exposure to AFB1 at 96 hpf. In contrast, at 96 hpf, *Hericium* was able to counteract the oxidative augmentation of the CAT, SOD, and GST levels. Our results demonstrate the ability of *Hericium* to inhibit alterations to the oxidative stress pathway. These data from the different changed activities of these antioxidant enzymes suggest that AFB1 stimulates and causes irreversible damage to the antioxidant system of zebrafish in the early stages of life, which is in accordance with a previous study on pacific white shrimp. Our data suggest that the presence of Hericium prevented alteration of the antioxidant balance during AFBI exposure. Similar results were also observed in zebrafish where the homeostatic maintenance of oxidative stress was also disrupted in the gut and liver after exposure to model pollutants with varied modes of action for 7 days [42].

Lipid peroxidation increased following AFB1 exposure, which may be able to be attributed to the induction of ROS, which enhances the oxidation of poly-unsaturated fatty acids and leads to lipid peroxidation [43]. AFB1 induced oxidative stress and led to oxidative damage with increased lipid peroxidation. Moreover, in these zebrafish, the basal levels of MDA were restored in the *Hericium* treatment group.

Increased ROS production associated with decreased antioxidant defense expression are not the only involved in the effects of AFB1 toxicity on zebrafish embryonic development, but apoptosis and the caspase pathway play also an important role [44]. Apoptosis is a regulated cell death mechanism and has a key role in several physiological and pathological processes [45]. ROS over-production has been shown to be an important apoptotic signal [46,47,48]. The induction of apoptosis in zebrafish embryos in vivo was detected by TUNEL, an abnormal apoptotic signal that was observed in the trunk, yolk, and tail after AFB1 exposure. Previous studies have suggested that metal ion-induced ROS production acts directly on the mitochondria to cause cytochrome c release from mitochondria into the cytosol, which leads to caspase-9 activation and apoptosis [49]. Caspases are a large family of proteinases that play crucial roles in the process of apoptosis and are considered markers of oxidative stress-induced apoptosis in zebrafish embryos [50]. Previous studies have demonstrated the ability of AFB1 to activate the intrinsic apoptotic pathway, which is normally regulated by the activation of caspase-9 [51]. In our study, exposure to AFB1 leads both, resulting in the induction of apoptosis accompanied by a significant increase in caspase-9 expression, which is in line with what has been determined previously [52]. The ability of *Hericium* to reduce AFB1-induced oxidative stress is also reflected in the decrease of the apoptotic process as well as a decrease of caspase-9 mRNA expression, which is often linked to increased ROS production.

## 4. Conclusions

In conclusion, exposure to AFB1, one of the mycotoxins that contaminates plant-based aquafeeds, at the concentration of 100 ng/mL on zebrafish embryos promoted malformation during larvae development. AFB1 strongly interferes with the development of the early life stages of zebrafish. Toxicity assays revealed that AFB1 induced dose-dependent mortality in zebrafish embryos, resulting in a LC50 value of 100 ng/mL after 96 h of exposure. This confirms that AFB1 has a detrimental effect on fish. AFB1 disrupted the normal morphology of embryonic zebrafish, and this condition is related to an increase in oxidative stress as well as to an augment of apoptosis. The assays conducted here indicate that AFB1-related increases of oxidative stress contributed to the detrimental effects of this mycotoxin on zebrafish embryos. Treatment with *Hericium erinaceus*, a known antioxidant compound derived from mushrooms, showed the ability to decrease the oxidative stress in zebrafish larvae after AFB1 exposure, and reduced apoptosis at the same, suggesting a potentially protective effect of this compound against mycotoxin intoxication.

## 5. Materials and Methods

### 5.1. Zebrafish Maintenance and Embryo Collection

Zebrafish maintenance and embryo collection from fertilized eggs were provided by the Center of Experimental Fish Pathology (Centro di ittiopatologia Sperimentale della Sicilia, CISS), University of Messina, Italy. Eggs were collected and selected within 4 h post fertilization (hpf) under a stereomicroscope (Leica M0205C, Multifocal). All embryos were derived from the same egg spawn.

### 5.2. Fish Embryo Toxicity (FET) Test

A fish embryo toxicity (FET) test was performed according to OECD [53] and ISO 15088. Zebrafish embryos exposed to AFB1 concentrations (10, 50, and 100 ng/mL) and *Hericium extract* (100 μg/L) in 5 mL of freshwater for 4–96 hpf were measured for toxic effects over a continuous observation period. The AFB1 and *Hericium* solutions were changed, and embryonic/larval mortality and hatching rate were assessed every 24 h. As we described in a previous paper [54], healthy embryos were placed in 24-well culture plates. Photographs of the embryos were obtained under a stereomicroscope (Leica M0205C, multifocal), and the percentage of abnormal embryos was assessed every 24 h. H. erinaceus biomasses were generously provided by Mycology Research Laboratories Ltd. (MRL, Luton, UK), and were used as received for the investigations. Hericium alcoholic extract was obtained as previously described [18].

### 5.3. Viability and Morphology after AFB1 Exposure and Hericium Protective Effect

AFB1 (CAS:1162-65-8) was purchased from the Sigma-Aldrich Company (St. Louis, MO, USA) and was first dissolved in DMSO at a concentration of 6 mg/mL and was stored at 20 °C as a stock solution. To examine the survival rate and morphology of the embryos/larvae, AFB1 in stock solution was diluted with 0.01M phosphate-buffered saline (PBS, pH7.0) to 0.3 mg/mL and was further diluted with embryo medium (15 mM NaCl, 0.5 mM KCl, 1 mM CaCl2, 1 mM MgSO4, 0.15 mM KH2PO4, 0.05 mM Na2HPO4, 0.7 mM NaHCO3, and a pH 7.3) to the tested concentrations (10, 50, and 100 ng/mL). Healthy and normally developing WT embryos were collected at 6 h post-fertilization (hpf) and were exposed to vehicle or various concentrations of AFB1 in embryo medium. Healthy embryos were collected at 6 h post-fertilization (hpf) and were exposed to various concentrations of AFB1 in embryo medium. The embryonic development was monitored, and parameters such as mortality, heartbeat, and hatching rate as well as potential malformations such as pericardial edema, pigmentation, and axial spinal curvature in the hatched larvae were evaluated during the exposure period [55]. To measure heart rate, embryos at 48, 72, and 96 hpf were moved to room temperature conditions and were allowed to stabilize for 30 min prior to manual counting. For each treatment condition, ten embryos were selected at random, and their heart rates were measured for four intervals of 20 s under a stereomicroscope.

### 5.4. Determination of MDA and Oxidative Stress after AFB1 Exposure and Hericium Antioxidant Effect

The 20 larvae fish from each beaker were defrosted and homogenized on ice with 180 μL ice-cold physiological saline. The homogenate was centrifuged at 4000× *g* at 4 °C for 15 min to obtain the supernatant. The content of MDA and GSH, SOD, CAT, GPX, and GST in the supernatant was analyzed using commercial kits (Nanjing Jiancheng Bioengineering Institute, Nanjing, China) as described previously [39,40,56,57,58].

### 5.5. TUNEL

TUNEL staining protocol was conducted in agreement with the manufacturer, Roche. The larval sections included in the paraffin were deparaffinized in xylene and were rehydrated by a series of alcohols at decreasing percentages of ethanol, permeabilized with 0.1 M citrate buffer, and then incubated in TUNEL reaction mixture for 60 min at 37 °C in the dark. The tissue was then rinsed in PBS three times for 5 min and was then observed using exciting wavelengths in the range of 520–560 nm (maximum 540; green) and in the range 570–620 nm (maximum 580 nm; red).

### 5.6. RNA Isolation and RT-PCR Analysis

Total RNA was isolated from the 15 zebrafish larvae using RNeasy Mini Kit (Qiagen, Milan, Italy) according to manufacturer protocols. RNA was then quantified using a Nanodrop Spectrometer, and subsequently, an equal quantity of RNA for each sample was used for cDNA synthesis using an iScriptTM cDNA Synthesis Kit (Bio-Rad, Milano, Italy) according to manufacturer protocols. iQTM SYBR Green Supermix (Bio-Rad, Milano, Italy). Real-time PCR was performed using a Bio-Rad CFX Real-Time PCR (Bio-Rad, Milano, Italy) Detection System with specifically designed primers for larval zebrafish, as described previously [44].

Caspase-9 mRNA

(Forward: AAATACATAGCAAGGCAACC, Reverse: CACAGGGAATCAAGAAAGG);

b-actin mRNA

(Forward: AAGTGCGACGTGGACA, Reverse: GTTTAGGTTGGTCGTTCGTTTGA).

Fold change in mRNA level was determined using the −∆∆Ct data analysis method [59].

### 5.7. Materials

All compounds used in this study were purchased from Sigma-Aldrich Company Ltd.

### 5.8. Statistical Evaluation

All values in the figures and text are expressed as the mean ± standard error of the mean (SEM) of N number of animals. The results were analyzed by one-way ANOVA followed by a Bonferroni post hoc test for multiple comparisons.

## Data Availability

Not applicable.
